# Endothelin-1 Mediates Oxaliplatin Resistance via Activation of YAP Signaling in Colorectal Cancer

**DOI:** 10.32604/or.2025.064463

**Published:** 2025-11-27

**Authors:** Ranran Yang, Dan Yuan, Chaohan Liang, Siying Zhu, Jie Huang, Yingqi Zhang, Weiling He, Qinghai Li, Hong Zhang

**Affiliations:** 1 Department of Gastrointestinal Surgery, The First Affiliated Hospital, Sun Yat-sen University, Guangzhou, 510080, China; 2 Institute of Precision Medicine, The First Affiliated Hospital, Sun Yat-sen University, Guangzhou, 510080, China; 3 Department of Biochemistry, Zhongshan School of Medicine, Sun Yat-sen University, Guangzhou, 510080, China; 4 School of Public Health, Sun Yat-Sen University, Guangzhou, 510080, China; 5 Department of Gastrointestinal Surgery, Xiang’an Hospital of Xiamen University, School of Medicine, Xiamen University, Xiamen, 361000, China

**Keywords:** Colorectal cancer, endothelin-1, β-arrestin1, yes-associated protein signaling, chemoresistance

## Abstract

**Background:**

Colorectal cancer (CRC) is a predominant contributor to global cancer-associated mortality worldwide. Oxaliplatin (OXP), a foundational chemotherapeutic agent for CRC, often exhibits limited efficacy due to the emergence of drug resistance. Although endothelin-1 (EDN1) has been implicated in tumor drug resistance, its role in oxaliplatin resistance in CRC remains poorly defined. This work aimed to define how EDN1 contributes to oxaliplatin resistance and to explore its potential as a therapeutic target.

**Methods:**

Public genomic datasets were analyzed to confirm EDN1 upregulation in colorectal cancer (CRC) and its association with poor prognosis. EDN1 expression was modulated in parental and oxaliplatin-resistant CRC cell lines via shRNA knockdown and lentiviral overexpression. Functional assays, including drug sensitivity, flow cytometry, and 5-Ethynyl-2^′^-deoxyuridine (EdU) proliferation, were conducted to assess resistance. Mechanistic studies employed dual-luciferase reporter assays, Western blotting, co-immunoprecipitation, and immunofluorescence. CRC-derived subcutaneous xenograft models were used to evaluate the therapeutic efficacy of EDN1 targeting *in vivo*.

**Results:**

The study identifies EDN1 as a pivotal mediator of oxaliplatin resistance in CRC. EDN1 expression is markedly upregulated in oxaliplatin-resistant CRC cells and is significantly associated with poor patient survival outcomes. Mechanistically, EDN1 overexpression activates the Yes-associated protein (YAP) signaling by promoting the nuclear translocation of β-arrestin1 (β-arr1), thereby facilitating chemoresistance. Importantly, the combinatorial inhibition of EDN1, in conjunction with oxaliplatin treatment, substantially enhances apoptosis and suppresses tumor growth both *in vitro* and *in vivo*.

**Conclusion:**

The study demonstrates that EDN1 governs oxaliplatin resistance through the β-arr1/YAP axis and provides preclinical evidence for targeting EDN1 to overcome chemoresistance in CRC.

## 1 Introduction

Colorectal cancer (CRC) ranks among the most prevalent malignancies worldwide and remains a leading contributor to cancer-related mortality, with over 19.9 million new cases and 9.35 million fatalities annually [1]. Despite advances in multimodal therapies, including surgery, radiotherapy, and chemotherapy, the 5-year survival rate for metastatic CRC remains below 15% [2]. Oxaliplatin (OXP), a third-generation platinum-based chemotherapeutic, has revolutionized CRC treatment as a key component of the FOLFOX regimen (5-fluorouracil [5-FU], leucovorin, and oxaliplatin) [3]. However, intrinsic or acquired resistance to oxaliplatin develops in >60% of patients, leading to disease progression and mortality [4–6]. Unraveling the molecular mechanisms underlying oxaliplatin resistance is thus imperative to devise strategies that restore therapeutic efficacy.

The endothelin (ET) axis, comprising three ligands (ET-1, ET-2, ET-3) and two G protein-coupled receptors (ETAR and ETBR), is a critical regulator of tumor progression and therapy resistance [7]. Endothelin-1 (EDN1), the most potent isoform, is a key regulator of vasoconstriction and has been well documented in cardiovascular and renal diseases [7]. Aside from its function as a powerful endogenous vasoconstrictor and mediator in renal and cardiovascular diseases, ET-1/ETAR signaling drives oncogenic pathways, including MAPK, PI3K/AKT, and PKC, to promote cell proliferation, growth, and survival [8–10]. While clinical trials targeting ET-1 in prostate cancer (e.g., zibotentan) failed to improve survival [11,12]. Recent studies highlight its role in chemoresistance. For example, ET-1 overexpression induces resistance to EGFR tyrosine kinase inhibitors in non-small cell lung cancer (NSCLC) by reducing tumor drug delivery via vascular remodeling [13]. ET-1 engages β-arrestin1 (β-arr1), a multifunctional scaffolding protein that integrates signaling crosstalk between GPCRs, receptor tyrosine kinases, and transcriptional regulators [14–16]. In ovarian cancer, β-arr1 mediates ETAR-driven Wnt/β-catenin activation and YAP nuclear translocation [17]. Tocci et al. further demonstrated that ET-1/β-arr1/RhoA signaling activates YAP/TEAD transcription, enabling apoptosis evasion [18]. Despite these advances, the role of the ET axis in oxaliplatin resistance, particularly in CRC, remains unexplored.

Yes-associated protein (YAP) and transcriptional co-activator with PDZ-binding motif (TAZ), central effectors of the Hippo pathway, are increasingly implicated in chemoresistance [19,20]. YAP/TAZ activation is regulated not only by Hippo kinases but also by GPCRs, including ETAR, through Rho GTPase-dependent cytoskeletal remodeling [21–23]. In CRC, YAP hyperactivation correlates with resistance to 5-FU [24] and cetuximab [25], as well as a poor prognosis. While YAP’s role in ET-1-mediated resistance has been characterized in ovarian cancer [18]. Its interplay with the ET axis in CRC—and specifically in oxaliplatin resistance—remains undefined.

Here, the study aims to investigate the central regulatory role of EDN1 in oxaliplatin resistance in CRC and its underlying molecular mechanisms. By integrating *in vitro* and *in vivo* models, the research focuses on elucidating the specific pathway through which oxaliplatin-induced EDN1 overexpression triggers β-arr1 nuclear translocation and subsequent YAP activation. It further seeks to unravel the molecular basis of the interaction between nuclear β-arr1 and YAP in enhancing transcriptional activity, particularly its regulation of pro-survival genes and anti-apoptotic effects. Additionally, the study evaluates the efficacy of the endothelin receptor antagonist bosentan in targeting EDN1 signaling to reverse chemoresistance and systematically validates the therapeutic potential of the EDN1/β-arr1/YAP axis as a combinatorial strategy to overcome chemotherapy resistance in CRC. The ultimate goal is to provide a theoretical foundation for developing precision combination therapies targeting this molecular axis in clinical settings.

## 2 Materials and Methods

### 2.1 Cell Culture

Human colorectal cancer cell lines HCT8, HT29, SW480, LoVo, SW620, SW837, DLD1 and 293T were acquired from ATCC (Manassas, VA, USA). HCT8/L (MXC508, Shanghai, China) was obtained from the Shanghai Meixuan Biotechnology Co., Ltd., and HCT116/L (CTCC-0313-NY, Jinhua, China) was obtained from Zhejiang Meisen Cell Technology Co., Ltd. All cells were identified by Short Tandem Repeat (STR) and there was no risk of mycoplasma infection. HCT8, HT29, DLD1 and HCT8/L were cultured with RPMI 1640 (Gibco, 6124511, Suzhou, China) medium containing 1% antibiotics Penicillin Streptomycin (Gibco, 15140-122, Grand Island, NE, USA) and 10% fetal bovine serum (Gibco, A52567, Grand Island, NE, USA). SW480, LoVo, SW620, SW837 and HCT116/L were cultured with DMEM (Gibco, 6124644, Suzhou, China) medium containing 1% antibiotics, Penicillin Streptomycin and 10% fetal bovine serum. These cells were cultured in a 37°C cell culture incubator with 5% CO_2_.

### 2.2 Animal Experiments

Ethical approval for all *in vivo* studies was obtained from the Institutional Animal Ethics Committee of Sun Yat-sen University (Guangzhou, China). The study complied with all relevant ethical regulations regarding animal research (ethical approval code: SYSU-IACUC-2023-000365). 5–6 weeks-old female thymus-free nude mice were purchased from Vitonlihua (SCXK (Beijing) 2021-0006, Beijing, China) and housed in the Laboratory Animal Facilities, Sun Yat-sen University. We constructed cell-derived subcutaneous xenograft (CDX) models to study the effects of combined treatment with the EDN1 inhibitor bosentan and oxaliplatin. A total of 5 × 10^6^ viable HT29 cells were injected subcutaneously into thymus-free nude mice, and tumor size was measured with vernier calipers until it reached 80 mm^3^. Mice were randomly divided into four groups of eight mice each. They were given the following treatments: vectors (5% DMSO, 40% PEG300, 5% Tween-80, 50% saline, and 5% W/V Glucose Solution), bosentan (100 mg/kg), oxaliplatin (5 mg/kg) oxaliplatin (5 mg/kg) and bosentan (100 mg/kg), respectively. Oxaliplatin was administered intraperitoneally every three days, and bosentan was administered daily by gavage.

Tumor length and width were measured every three days, and tumor volume was calculated as 0.5 × D × W^2^. Upon completion of the experiment, the mice were euthanized in a humane manner, and the tumors underwent removal, photography, weighing, and embedding in paraffin for subsequent pathological examination.

### 2.3 Bioinformatics Analysis

The pan-cancer RNA-seq integrated dataset (the Cancer Genome Atlas (TCGA), TARGET, Genotype-Tissue Expression (GTEx), sample size *N* = 19,131) was obtained from the UCSC Xena platform (https://xenabrowser.net, accessed on 07 April 2025). This dataset provides standardized gene expression data, including that of ENSG00000078401 (EDN1). For the specific analysis of CRC, the TCGA Colon and Rectal Cancer cohort data (*N* = 434) were additionally retrieved from the same platform. The expression level of EDN1 was quantified in transcripts per million (TPM) and normalized by Log_2_[TPM + 1] transformation, followed by filtering out genes with a mean TPM < 1. Differential expression of EDN1 between tumor and normal tissues was analyzed using the ‘DESeq2’ package (v1.40.2), and the results were visualized using ‘ggplot2’ (v3.4.4). The GSE103479 dataset in the Gene Expression Omnibus database (GEO) has been further integrated. Patients were divided into high and low expression groups based on the median expression of EDN1. Kaplan-Meier survival analysis was performed using the ‘survival’ package (v3.5-7), and the survival curves were plotted using ‘ggplot2’. To explore the mechanism of oxaliplatin resistance, the GSE42387 dataset was used, which includes parental strains (HCT116, LOVO, HT29) and their oxaliplatin-resistant strains. Differential genes were screened using ‘DESeq2’ (threshold: |Log_2_FC| > 1, FDR < 0.05) for each group. Intersecting genes among the three groups were identified using Venn diagrams. A heatmap was generated using the ‘pheatmap’ package (v1.0.12) to display the clustering tree of samples and to annotate core genes such as EDN1.

### 2.4 Analysis of Oxaliplatin Resistance

To investigate the correlation between oxaliplatin resistance and EDN1 mRNA expression, we evaluated oxaliplatin sensitivity by determining IC_50_ values and quantified EDN1 mRNA expression across seven colorectal cancer cell lines (HCT8, SW620, SW480, SW837, DLD1, LoVo, HT29). First, the IC_50_ values of each cell line were normalized using Z-score transformation to eliminate inter-cell line variability. Cells were then classified into high-expression (EDN1 High) and low-expression (EDN1 Low) groups based on the median EDN1 mRNA expression level. Finally, the correlation between IC_50_ values and EDN1 mRNA expression was statistically evaluated to determine potential associations.

### 2.5 Immunofluorescence

3 × 10^4^ HCT8 cells and HT29 cells were grown in a 24-well plate and treated with oxaliplatin (10 μM, 48 h), bosentan (40 μM, 24 h), or a combination of both drugs. It was first fixed using 4% paraformaldehyde and permeated with 0.25% Triton X-100 (Beyotime, ST797, Shanghai, China). Bovine Serum Albumin (BSA) was incubated for 2 h and then incubated with β-arrestin1 antibodies (Abcam, ab32099, 1:150, Fremont, CA, USA) overnight. The nuclei were incubated for 1 h with Alexa Fluor™ 488 (Cell Signaling Technology, 4412S, Danvers, MA, USA) protected from light and stained with 4^′^,6-diamidino-2-phenylindole (DAPI) (Beyotime, C1006, Shanghai, China). The final images were captured with a laser scanning confocal microscope (Nikon Eclipse Ni-E, Tokyo, Japan).

### 2.6 Immunohistochemical Analysis

The standard techniques were employed for conducting immunohistochemistry. Dewaxing and rehydration of paraffin-embedded sections were performed. After placing the slices in the autoclave, the antigen was repaired for 20 min at medium-high heat with Ethylene Diamine Tetraacetic Acid (1.0 mM EDTA) and allowed to cool naturally to room temperature. Endogenous peroxidase activity was inhibited for 30 min using 3% H_2_O_2_. Paraffin section samples that have undergone antigen repair were sealed with blocking solution (Beyotime, P0102, Shanghai, China) for 30 min at room temperature to prevent non-specific binding. Subsequently, the sections were incubated overnight at 4°C with the primary antibodies Ki67 (ZSGB-BIO, ZM-0167, 1:500, Beijing, China) and Cleaved Caspase-3 (Cell Signaling Technology, 9661, 1:400, Danvers, MA, USA), followed by an incubation with a biotinylated secondary antibody (ZSGB-BIO, PV-9000, Beijing, China) at room temperature for 1 h. The color was displayed using 3,3^′^-Diaminobenzidine (1 × DAB) (ZSGB-BIO, ZLI-9018, Beijing, China) and retouched with Harris Retouch Hematoxylin (Solarbio, G1120, Beijing, China). Finally, the sections were scanned and imaged using a slide scanner (KFBIO, KF-PRO-020, Ningbo, China).

### 2.7 Construction of Knockdown Overexpression Plasmids & Plasmid Sequences

The delivery of short hairpin RNA (shRNA) (Tsingke, Beijing, China) via lentivirus was used to achieve stable knockdown of target genes. The primer sequences of pLKO.1 Vectors constructed with anti-puromycin plasmids are shown in Table 1.

**Table 1:** The shRNA primer sequence used in this study

Construct	Species	Direction	Sequence (5^′^-3^′^)
shEDN1-1	Human	Forward	CCGGGCTCGTCCCTGATGGATAAAGCTCGAGC TTTATCCATCAGGGACGAGCTTTTTG
		Reverse	AATTCAAAAAGCTCGTCCCTGATGGATAAA GCTCGAGCTTTATCCATCAGGGACGAGC
shEDN1-2	Human	Forward	CCGGGCCCTCCAGAGAGCGTTATGTCTC GAGACATAACGCTCTCTGGAGGGCTTTTTG
		Reverse	AATTCAAAAAGCCCTCCAGAGAGCGTT ATGTCTCGAGACATAACGCTCTCTGGAGGGC
negative control	Human	Forward	CCGGCTCGAGTTTTTG
		Reverse	AATTCAAAAACTCGAG

In the EDN1 overexpression system, the EDN1 cDNA (NM_001168319.2) was cloned into the pLVX-IRSE-Puro lentiviral vector (Fenghbio, BR025, Changsha, China). To perform knockdown and overexpression experiments, the pLKO.1 and pLVX constructs, along with packaging and helper plasmids PAX2 and MD2G, were co-transfected into 293T cells using the Lipo8000 transfection reagent (Beyotime, C0533, Shanghai, China). The virus was harvested, filtered, and titrated prior to infecting target cells with 10 mg/mL of polybrene (Solarbio, H8761, Beijing, China). Subsequently, puromycin was used to screen infected cells accordingly.

### 2.8 Western Blot & Antibodies

Human CRC cell lines (HT29, HCT8, SW480, and LoVo) were seeded in six-well plates (3 × 10^5^ cells/well). After a suitable period of dosing, 80–100 μL of RIPA Lysis Buffer (Beyotime, P0013, Shanghai, China) containing protease inhibitor (Sigma, P8340, St. Louis, MO, USA) and phosphatase inhibitor (Sigma, P0044, St. Louis, MO, USA) was added for complete lysis. The supernatant was removed by centrifugation for 20 min at 4°C. Total protein levels were assessed with a BCA assay kit (Beyotime, China; Cat# P0011). The cytoplasmic and nuclear proteins were separated and extracted according to the instructions in the NE-PER^TM^ Nuclear and Cytoplasmic Extraction Reagents (Thermo Fisher Scientific, 78833, Waltham, MA, USA).

Protein samples were separated by 10% SDS-PAGE and then transferred onto polyvinylidene fluoride (PVDF) membranes (Millipore, IPVH00010, Burlington, MA, USA). The membranes were blocked with 10% skimmed milk for 1 h at room temperature, followed by overnight incubation at 4°C with primary antibodies detailed in Table 2. Subsequently, the membranes were incubated for 1 h at room temperature with a secondary antibody (Abcam, ab6702, Cambridge, UK). Protein signals were detected using an ECL chemiluminescence kit (Fdbio Science, FD8000, Hangzhou, China) on an imaging system (Tanon, 4600, Shanghai, China). Table 2 lists all the antibodies used in this study.

**Table 2:** The information related to the antibodies used in this study

Target	Application	Dilution ratios	Vendor	Catalog number
ERK1/2	WB	1:1000	CST	4695
p-ERK1/2	WB	1:1000	CST	4370
P38	WB	1:1000	CST	54470
p-P38	WB	1:1000	CST	4511
YAP	WB/IP	1:1000/1:100	CST	14074
p-YAP(Tyr357)	WB	1:1000	Abcam	ab62751
β-arrestin1	WB/IF/IP	1:1000/1:150/1:200	Abcam	ab32099
Lamin B	WB	1:1000	CST	13435
Cleaved Caspase-3	IHC	1:400	CST	9661
α-tubulin	WB	1:20,000	Proteintech	66031-1-lg
Vinculin	WB	1:1000	CST	13901
GAPDH	WB	1:10,000	Bioworld	AP0063
Ki67	IHC	1:500	ZSGB-BIO	ZM-0167
P21	WB	1:1000	CST	2947S
EDN1	WB	1:500	Affinity	DF6126

Note: WB, Western blot; IHC, Immunohistochemistry; CST, Cell Signaling Technology; IF, immunofluorescence.

### 2.9 Enzyme-Linked Immunosorbent Assay (ELISA)

Detection was performed using an ET-1 ELISA Kit (Huamei, CSB-E07007h, Wuhan, China). On six-well plates, 1 × 10^6^ HCT8/L cells and HCT116/L cells were grown and allowed to adhere for 12 h. The supernatant was collected and then subjected to centrifugation at 1000× *g* for 5 min at 4°C (Eppendorf, 5425R, Hamburg, Germany) to remove the sediment, and stored at −80°C while the cells were counted (Countstar, Countstar Rigel S5, Shanghai, China). EDN1 secretion levels were analyzed and normalized by cell count.

### 2.10 Luciferase Reporter Gene Assay

The dual luciferase reporter assay system (Promega, E1910, Madison, WI, USA) was utilized to measure the luciferase activity, in accordance with the manufacturer’s instructions. Renilla (internal control), luciferase reporter plasmids NF-kB, P53, and the YAP promoter reporter plasmid were transiently transfected using Lipo8000 transfection reagent (Beyotime, C0533, Shanghai, China). The firefly and sea kidney luciferase activities were measured as previously described [26]. Data are expressed as the mean ± standard deviation.

### 2.11 Apoptosis

The Annexin V-AF647/PI Apoptosis Detection Kit (GOONIE, AP006-100, Hangzhou, China) was used to assess apoptosis. 8 × 10^5^ HT29 cells were evenly inoculated in small 60 mm^2^ dishes, and the corresponding concentrations of oxaliplatin (20 μM, MCE, HY-17371, Shanghai, China) were added separately. The cell supernatant was collected after 48 h of drug action, and the cells were digested using EDTA-free trypsin before being washed with pre-cooled 0.01M PBS (pH7.3 ± 0.1). The cell precipitate was resuspended in binding buffer, prepared by diluting the 5× stock with PBS. The cell precipitates were stained for 10 min using 10 μL PE and 5 μL Annexin V-AF647. Then we added 400 μL of 1× binding buffer and immediately mounted the machine (Agilent, Agilent NovoCyte, Palo Alto, CA, USA) for flow-through apoptosis detection.

### 2.12 5-Ethynyl-2^′^-Deoxyuridine (EdU) Assay for Cell Proliferation

EdU test was performed using the BeyoClick EdU-555 or EdU-488 Cell Proliferation Assay Kit (Beyotime, C0075S, C0071S, Shanghai, China). 2 × 10^5^ HT29 and HCT8 cells were cultured in 12-well plates and treated with BeyoClick EdU-555 or EdU-488 Cell Proliferation Assay Kit [27]. We observed the labeled cells using a fluorescent microscope (Zeiss, Axio Observer 7, Oberkochen, Germany) as Alexa Fluor 555-positive cells (red) or EdU-488 cells (green) were proliferating cells, and Hoechst 33342 (blue) stained the nucleus.

### 2.13 Cell Proliferation Assay and Cytotoxicity Assays

1 × 10^4^ HT29 cells were grown in each well of a 12-well plate, and cell value was measured by daily counts for seven days. IC_50_ values were detected using the Cell Counting Kit-8(CCK8) (GLPBIO, GK10001, California, USA). A total of 5000 cells were seeded per well; 3 replicate wells were required for each drug concentration. Dosing was carried out on the second day with a gradient of oxaliplatin concentrations of 0, 0.05, 0.25, 0.5, 1, 2, 5, 10, 20, and 32 μg/mL. CCK8 was added 48 h after dosing to determine the IC_50_ value. The CCK8 to medium ratio was 1:9, and the values were determined by absorbance at 450 nm in a microplate reader (Thermo Fisher, Thermo Fisher Varioskan LUX, Waltham, MA, USA) after 2 h of incubation at 37°C in an incubator protected from light [28].

Total RNA was extracted using the RNA Quick Purification Kit (ESscience, RN001, Shanghai, China) based on the manufacturer’s guidelines. Reverse transcription was performed using the Prime Script RT Kit (Takara, RR036A, Kyoto, Japan) to synthesize cDNA. The Applied Biosystems QuantStudio 5 Real-Time PCR system (ThermoFisher Scientific, Carlsbad, CA, USA) was used to conduct real-time reverse transcription PCR with the aid of SYBR-Green Master Mix (Takara, RR820B, Kyoto, Japan). GAPDH was used as a normalized internal control. In Table 3, all primers employed in this research are enumerated (2^−ΔΔCt^).

**Table 3:** The primer sequence used in this study

Construct	Species	Direction	Sequence (5^′^-3^′^)
EDN1	Human	Forward	AGAGTGTGTCTACTTCTGCCA
	Human	Reverse	CTTCCAAGTCCATACGGAACAA
AKR1B10	Human	Forward	TCAGAATGAACATGAAGTGGGG
	Human	Reverse	TGGGCCACAACTTGCTGAC
GALNT5	Human	Forward	GGGCGAGTCTTGGCATTTATC
	Human	Reverse	CCCGAGTGTTGATCTCACTGAAT
NUPR1	Human	Forward	CTCTCATCATGCCTATGCCTACT
	Human	Reverse	CCTCCACCTCCTGTAACCAAG
VIM	Human	Forward	GACGCCATCAACACCGAGTT
	Human	Reverse	CTTTGTCGTTGGTTAGCTGGT
SLC43A1	Human	Forward	GGACGTGGAAGCTCTGTCTC
	Human	Reverse	GCAGCGTGAGTGAAGTGAAC
TMEM173	Human	Forward	CCAGAGCACACTCTCCGGTA
	Human	Reverse	CGCATTTGGGAGGGAGTAGTA
SLC39A8-	Human	Forward	ATGCTACCCAAATAACCAGCTC
	Human	Reverse	ACAGGAATCCATATCCCCAAACT
SNTB1	Human	Forward	CTGGAAGTGAAGTACATGCGAG
	Human	Reverse	GGGATGCTTTTCCGGTCTCTG
AKR1C3	Human	Forward	GTCATCCGTATTTCAACCGGAG
	Human	Reverse	CCACCCATCGTTTGTCTCGTT
AMIGO2	Human	Forward	CCTGGGAACCTTTTCAGACTG
	Human	Reverse	GCAAACGATACTGGAATCCACT
GAPDH	Human	Forward	GCACCGTCAAGGCTGAGAAC
	Human	Reverse	TGGTGAAGACGCCAGTGGA

### 2.14 Co-Immunoprecipitation (Co-IP) Protocol

293T cells treated with 10 μM oxaliplatin for 24 h were lysed in RIPA buffer (Fdbio Science, FD011, Hangzhou, China) supplemented with protease inhibitors (Sigma, P8340, St. Louis, MO, USA) for 30 min on ice (4°C with gentle agitation). Total protein lysates were obtained by centrifugation at 12,000× *g* for 15 min at 4°C, with 2% of the lysate reserved as input controls. The remaining lysates were incubated overnight at 4°C with either anti-YAP antibody (1:100 dilution, Cell Signaling Technology, 14074T, Danvers, MA, USA) or species-matched normal IgG (1:100 dilution, Cell Signaling Technology, 2729, Danvers, MA, USA). Protein A/G-agarose beads (Santa Cruz Biotechnology, sc-2003, Dallas, TX, USA) were then added for 4 h at 4°C with rotation. After five washes with PBS containing 1 mM Phenylmethanesulfonyl fluoride (PMSF) (Beyotime, ST507, Shanghai, China), bound proteins were eluted in protein loading buffer (Fdbio Science, FD001, Hangzhou, China) at 95°C for 10 min. Input controls and IP eluates were analyzed by immunoblotting using standard protocols.

### 2.15 Statistical Analysis

The software of GraphPad Prism (Boston, NY, USA) was utilized to calculate means, standard deviation (SD), and standard error of mean (SEM). Statistical differences between the specified groups were compared using a two-tailed Student’s *t*-test. Pearson correlation analysis was conducted to examine the correlations between genes. Tukey’s and Šídák’s multiple comparisons tests were used for statistical analysis of variance within multiple groups. Two-way ANOVA followed by Dunnett’s post hoc test was used to evaluate differences across multiple groups. A *p*-value < 0.05 was considered statistically significant.

## 3 Results

### 3.1 Elevated EDN1 Expression Correlates with CRC Progression and Poor Prognosis

**Figure 1:**
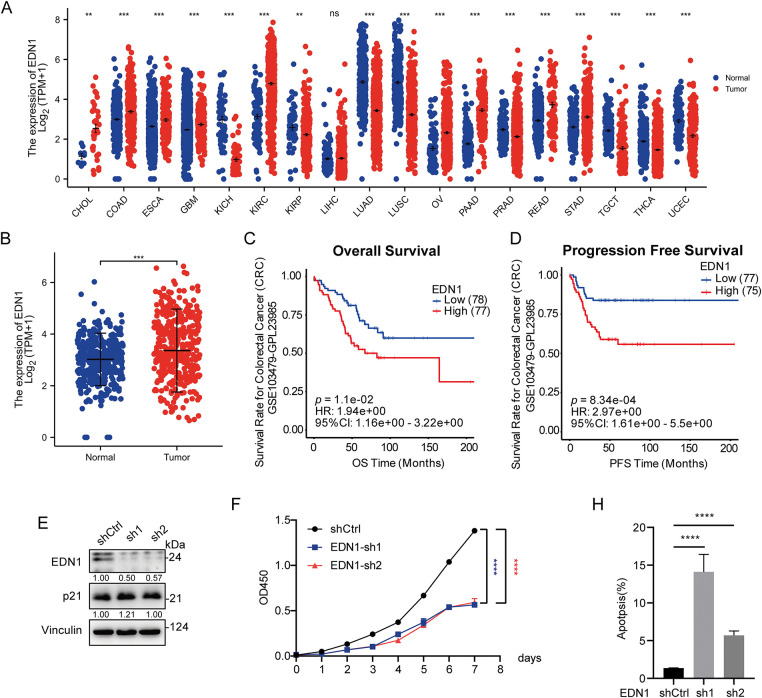

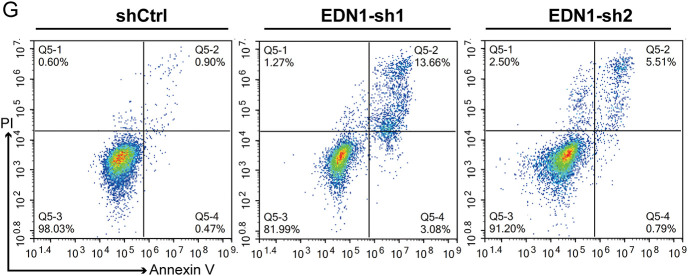
Increased EDN1 expression correlates with CRC progression. (**A**) EDN1 mRNA expression between tumor and normal tissues in 18 cancer types in the TCGA datasets. (**B**) EDN1 mRNA expression between tumor and normal tissues in the TCGA-CRC dataset. (**C**,**D**) Kaplan-Meier overall survival (**C**) and progression-free survival (**D**) curves of individuals with different EDN1 mRNA expression in the GEO-CRC dataset. (**E**) The efficacy of shRNA-mediated EDN1 knockdown in HT29 and the expression of p21 were evaluated by western blotting. (**F**) Cell proliferation in EDN1-knockdown cell lines. Assays were performed using the CCK8 kit. (**G**,**H**) Flow cytometry (**G**) and quantification analysis (**H**) with Annexin V/PI staining evaluated the proportions of viable (Annexin V^−^/PI^−^), early apoptotic (Annexin V^+^/PI^−^), and late apoptotic cells among the control and EDN1-knockdown HT29 cells. Data are shown as mean ± SD of three replicates. ****p < 0.0001, ***p < 0.001, **p < 0.01, ns, no significance

To assess the oncogenic role of EDN1 across malignancies, we analyzed its expression in 18 cancer types using the TCGA dataset. EDN1 exhibited context-dependent dysregulation, with significant upregulation in 9 cancers (including CRC, gastric, and ovarian cancers) and downregulation in 9 others (e.g., breast, prostate) (Fig. 1A). In CRC, EDN1 mRNA levels were consistently elevated in tumor tissues compared to normal tissues (*p* < 0.001; Fig. 1B), highlighting its CRC-specific oncogenic potential. Clinically, high EDN1 expression correlated with reduced overall survival (OS; HR = 1.94e+00, *p* = 1.1e−02) (Fig. 1C) and progression-free survival (PFS; HR = 2.97e+00, *p* = 8.34e−04) (Fig. 1D) in CRC patients. This CRC-specific prognostic relevance suggests EDN1’s role is context-dependent and tissue-restricted.

To validate its functional significance in CRC, we silenced EDN1 using two independent shRNAs (shEDN1-1 and shEDN1-2), achieving 50% knockdown efficiency (Fig. 1E). EDN1 depletion suppressed proliferation by 40% (*p* < 0.001; Fig. 1F) and increased apoptosis by more than 5-fold (*p* = 0.001; Fig. 1G,H). Notably, EDN1 knockdown did not alter p21 expression (Fig. 1E), indicating its pro-survival effects are independent of p21-mediated cell cycle regulation. Collectively, these results identify EDN1 as a context-dependent oncogene that is selectively upregulated in CRC and drives tumor progression through proliferation and apoptosis resistance.

### 3.2 EDN1 Expression Inversely Correlates with Oxaliplatin Sensitivity in CRC Cells

**Figure 2:**
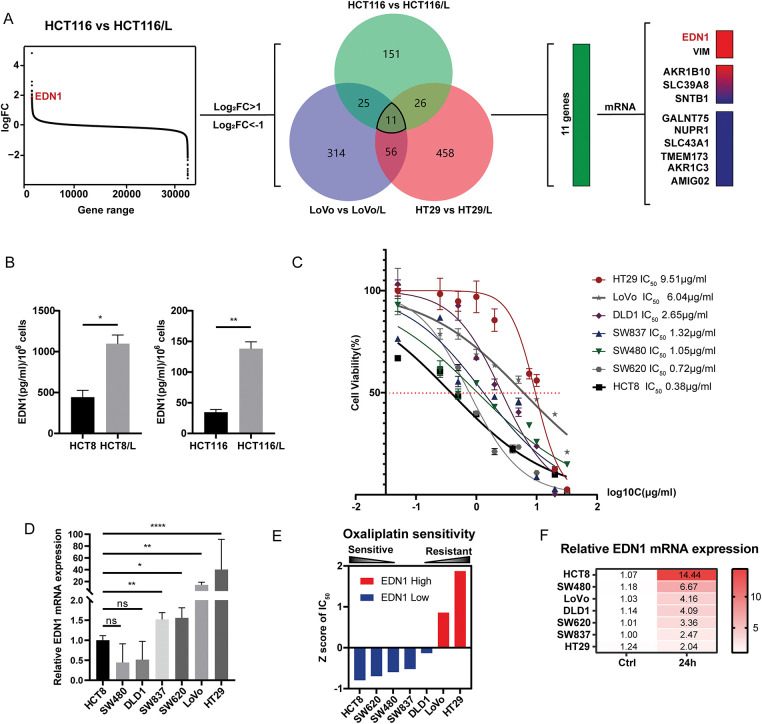

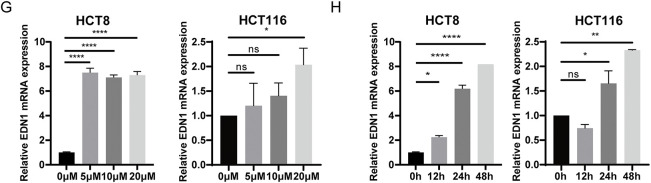
EDN1 expression levels negatively correlate with cellular oxaliplatin sensitivity. (**A**) Analysis of RNA-seq data results from three pairs of colorectal cancer cells and their corresponding oxaliplatin-resistant cells in the GEO database’s GSE42387 dataset. Heat map based on the relative mRNA expression of genes in three different oxaliplatin-resistant cells. (**B**) ELISA was performed to detect the protein content of oxaliplatin-sensitive and drug-resistant cell lines ET1. (**C**) Effect of oxaliplatin on the cell viability of seven colorectal cancer cell lines. Cells were assayed using CCK8 after 48 h of action using different oxaliplatin concentrations. (**D**) qPCR assays for relative mRNA expression levels of EDN1 against seven colorectal cancer cell lines. (**E**) The IC_50_ was normalized using the Z-score and grouped by relative EDN1 expression levels to analyze. (**F**) qPCR was performed to determine the relative expression levels of EDN1 mRNA in seven colorectal cancer cell lines after treatment with oxaliplatin (10 μM, 24 h). (**G**) EDN1 mRNA expression levels after treatment of HCT8 (left) and HCT116 (right) with different concentrations of oxaliplatin (48 h). (**H**) EDN1 mRNA expression levels after treatment of HCT8 (left) and HCT116 (right) with different times of oxaliplatin (10 μM). Data are shown as mean ± SD of three replicates. ****p < 0.0001, **p < 0.01, *p < 0.05, ns, no significance

**Figure A1:**
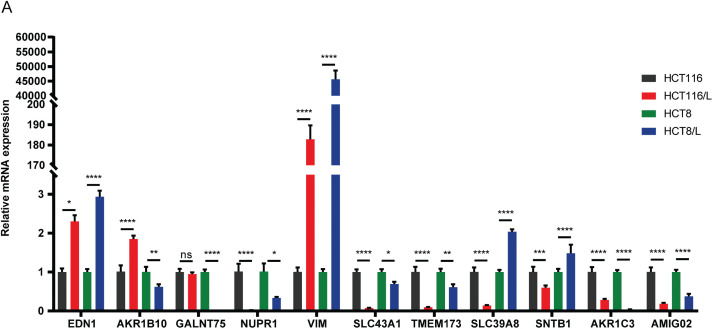

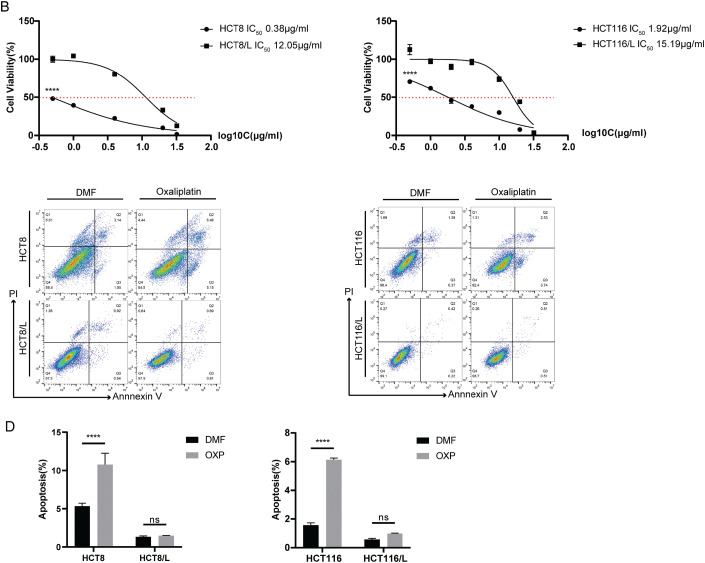
HCT8/L and HCT116/L were oxaliplatin resistant cells. (**A**) Relative mRNA expression levels of seven differentially expressed genes in two pairs of oxaliplatin-sensitive and oxaliplatin-resistant cells. (**B**) The effect of oxaliplatin on the cell viability of sensitive and resistant cells was measured using CCK8 after 48 h of treatment. (**C**) Flow cytometry was used to evaluate DMF or oxaliplatin (20 μM, 48 h) treatment of sensitive and resistant cells in terms of the percentage of live (Annexin V^−^/PI^−^), early apoptotic (Annexin V^+^/PI^−^) and late apoptotic (Annexin V^+^/PI^+^) cells. (**D**) Quantitative analysis of Annexin V/PI staining. The data are shown as the mean ± SD of three replicates. ****p < 0.0001, ***p < 0.001, **p < 0.01, *p < 0.05, ns, no significance.

To identify candidate drivers of oxaliplatin resistance in CRC, we analyzed RNA-seq data from the Gene Expression Omnibus (GEO) dataset GSE42387, which includes three pairs of CRC cell lines and their oxaliplatin-resistant derivatives (Fig. 2A). Differential expression analysis (*p*-values < 0.05, |log_2_FC| > 1) identified 1042 genes. Among these, 11 genes were consistently differentially expressed across all three resistant cells. Unsupervised clustering revealed EDN1 upregulation in three resistant lines compared to their parental cells (Fig. 2A). To validate these findings, we employed HCT8 and HCT116 cell lines alongside their oxaliplatin-resistant counterparts, HCT8/L and HCT116/L. It was observed that mRNA levels of EDN1 and VIM were increased in the resistant cells (Fig. A1A). Oxaliplatin resistance was confirmed by assessing half-maximal inhibitory concentration (IC_50_) values (Fig. A1B) and apoptosis rates following oxaliplatin treatment (Fig. A1C,D). ELISA further confirmed that EDN1 protein levels were elevated in resistant cells compared to their sensitive counterparts (Fig. 2B).

To explore the correlation between EDN1 expression and oxaliplatin sensitivity, we quantified oxaliplatin IC_50_ values (Fig. 2C) and EDN1 mRNA levels (Fig. 2D) across seven CRC cell lines. Based on IC_50_ values, HCT8 was classified as oxaliplatin-sensitive, while HT29 was identified as oxaliplatin-insensitive (Fig. 2C). IC_50_ values were normalized using Z-scores and stratified into EDN1 high and low expression groups based on mRNA levels. Strikingly, EDN1 expression inversely correlated with oxaliplatin sensitivity across seven CRC cell lines (Fig. 2E). Following treatment with OXP (10 μM, 24 h), a generalized elevation of EDN1 mRNA expression levels was observed in colorectal cancer (CRC) tumor cells, with OXP-sensitive cell lines (e.g., HCT8) exhibiting a more pronounced upregulation of EDN1 expression (Fig. 2F). We further performed dose- and time-course experiments in HCT8 and HCT116 cells to delineate the kinetics of EDN1 induction. EDN1 mRNA levels increased progressively with oxaliplatin concentration (5–20 μM) in HCT8 cells and no significant change in HCT116 (Fig. 2G). Similarly, prolonged exposure (0–48 h) to 10 μM oxaliplatin induced time-dependent EDN1 upregulation, reaching maximal expression at 48 h (Fig. 2H). These results demonstrate that EDN1 induction is both dose- and time-dependent, with pronounced upregulation in oxaliplatin-sensitive cells. The inverse correlation between basal EDN1 levels and oxaliplatin sensitivity further positions EDN1 as a critical mediator of adaptive chemoresistance in CRC.

### 3.3 EDN1 Overexpression Confers Oxaliplatin Resistance in CRC

**Figure 3:**
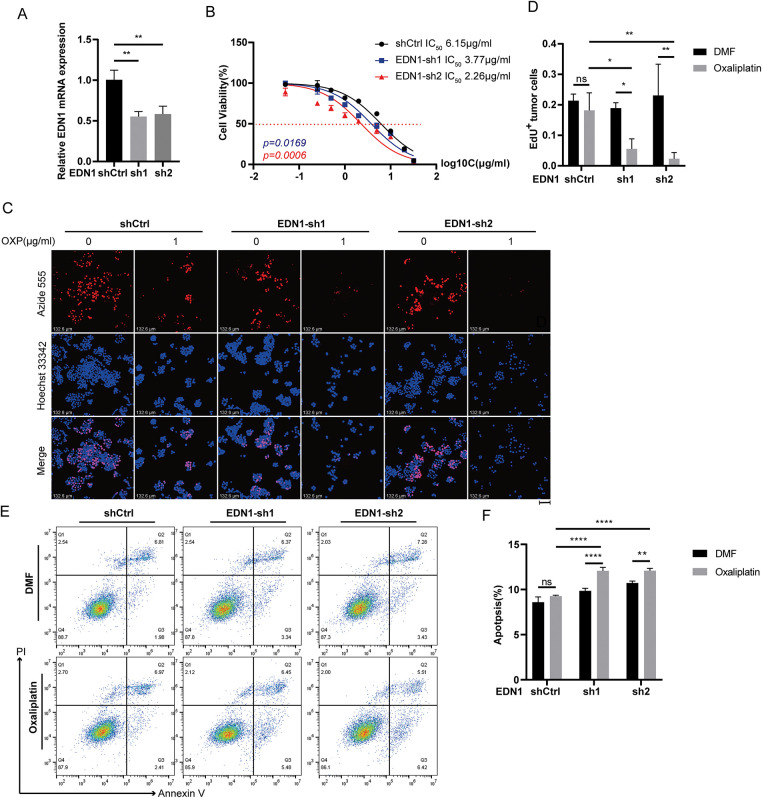
Effects on cells after silencing of EDN1 in oxaliplatin treatment-insensitive cell lines. (**A**) The efficiency of shRNA-mediated EDN1 knockdown in HT29 was assessed by qPCR. (**B**) Effect of oxaliplatin on the cell viability of the HT29 knockdown EDN1 cell line and control strain. Different concentrations of oxaliplatin were treated for 48 h and assayed using a CCK8 kit. (**C**) EdU assay of oxaliplatin-treated (10 μM, 48 h) EDN1-knockdown HT29 cell lines for proliferation. Representative images: Azide555 (red) represents cell proliferation, and Hoechst 33342 (blue) represents the nucleus. (**D**) Quantitative analysis of EdU assay results. (**E,F**) Flow cytometry (**E**) and quantification analysis (**F**) with Annexin V/PI staining evaluated the percentages of live cells (Annexin V^−^/PI^−^), early apoptotic cells (Annexin V^+^/PI^−^) and late apoptotic cells (Annexin V^+^/PI^+^) among the control and EDN1-knockdown HT29 cells treated with DMF or oxaliplatin (20 μM, 48 h). Data are shown as mean ± SD of three replicates. ****p < 0.0001, **p < 0.01, *p < 0.05, ns, no significance

**Figure 4:**
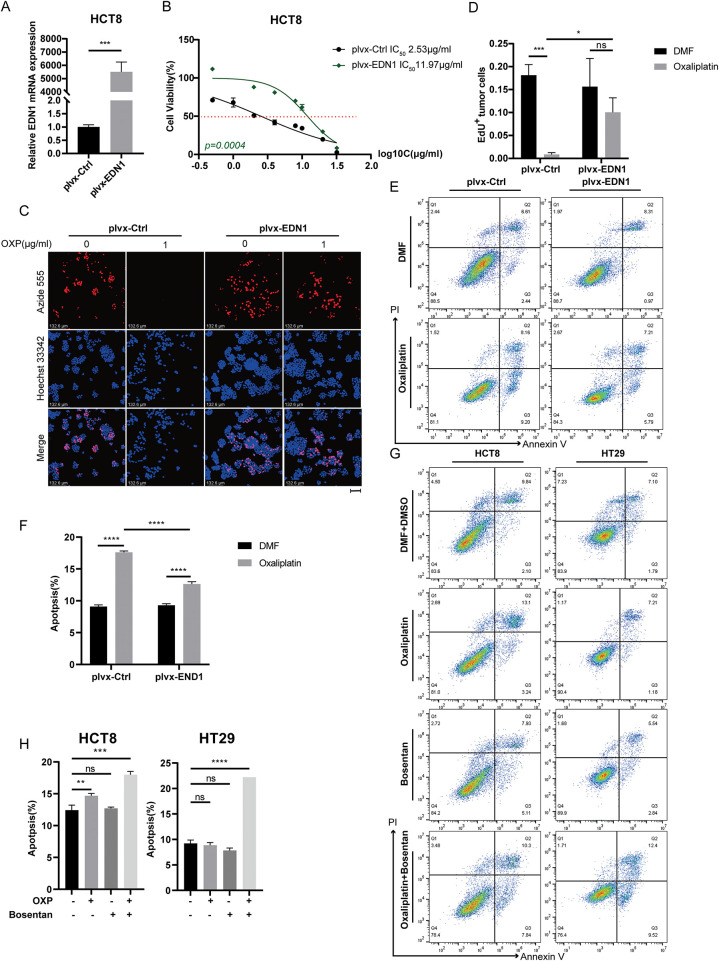
Overexpression of EDN1 promotes oxaliplatin resistance and reduces apoptosis *in vitro*. (**A**) The efficiency of EDN1 overexpression in HCT8 was assessed by qPCR. (**B**) Effect of oxaliplatin on the cell viability of the HCT8 overexpressing EDN1 cell line and control strain. Different concentrations of oxaliplatin were treated for 48 h and assayed using a CCK8 kit. (**C**) EdU assay of oxaliplatin-treated (10 μM, 48 h) HCT8 overexpressing cell lines for proliferation. Representative images: Azide555 (red) represents cell proliferation, and Hoechst 33342 (blue) represents the nucleus. (**D**) Quantitative analysis of EdU assay results. (**E,F**) Flow cytometry (**E**) and quantification analysis (**F**) with Annexin V/PI staining evaluated the percentages of live cells (Annexin V^−^/PI^−^), early apoptotic cells (Annexin V^+^/PI^−^) and late apoptotic cells (Annexin V^+^/PI^+^) among the control and HCT8 overexpressing cell lines treated with DMF or oxaliplatin (20 μM, 48 h). (**G**) Flow cytometry was used to evaluate bosentan (80 μM, 24 h) or oxaliplatin (20 μM, 48 h) treatment of HCT8 and HT29 cells in live cells (Annexin V^−^/PI^−^), early apoptotic cells (Annexin V^+^/PI^−^) and late apoptotic cells (Annexin V^+^/PI^+^) percentages. (**H**) Quantitative analysis of Annexin V/PI staining. Data are shown as a mean ± SD for three replicates. ****p < 0.0001, ***p < 0.001, **p < 0.01, *p < 0.05, ns, no significance

To investigate whether EDN1 overexpression confers oxaliplatin resistance, we stably overexpressed EDN1 in oxaliplatin-sensitive HCT8 cells and genetically silenced EDN1 in oxaliplatin-resistant HT29 cells. Successful EDN1 knockdown (Fig. 3A) and overexpression (Fig. 4A) were confirmed by qPCR. EDN1 overexpression in HCT8 cells markedly elevated the oxaliplatin IC_50_ (Fig. 4B), indicating enhanced chemoresistance. Conversely, EDN1 knockdown in HT29 cells resulted in a significant reduction in IC_50_ compared to controls (Fig. 3B), suggesting restored drug sensitivity.

Functional assessments by EdU staining demonstrated that EDN1 knockdown suppressed cellular proliferation in HT29 cells following oxaliplatin treatment (Fig. 3C,D), whereas EDN1 overexpression augmented proliferative capacity in HCT8 cells (Fig. 4C,D). Additionally, EDN1 overexpression attenuated oxaliplatin-induced apoptosis in HCT8 cells (Fig. 4E,F), while EDN1 knockdown significantly potentiated apoptosis in HT29 cells (Fig. 3E,F).

To explore potential therapeutic strategies, we investigated the combinatorial efficacy of oxaliplatin with bosentan, a dual endothelin receptor antagonist. Co-treatment synergistically enhanced apoptosis in both HCT8 and HT29 cells compared to oxaliplatin monotherapy (Fig. 4G,H). Collectively, these results demonstrate that EDN1 overexpression drives oxaliplatin resistance in CRC, while its depletion restores chemosensitivity, underscoring its pivotal role in oxaliplatin resistance.

### 3.4 EDN1 Drives Oxaliplatin Resistance in Colorectal Cancer via Activation of YAP Signaling

**Figure 5:**
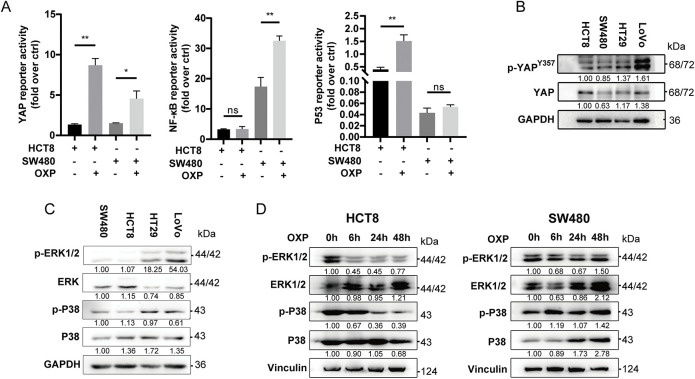

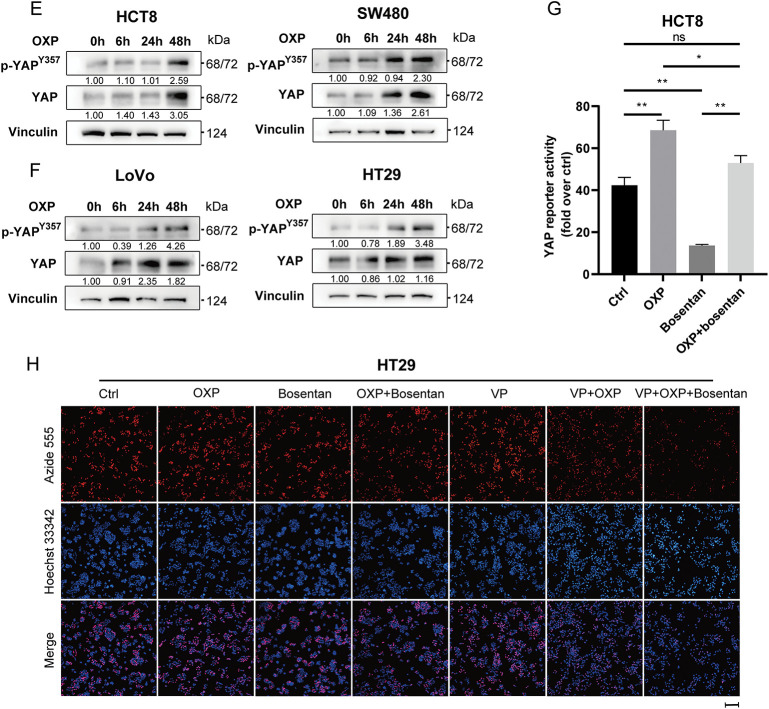
Oxaliplatin promotes the activation of YAP signaling pathway. (**A**) Transiently transfected YAP reporter, NF-kB reporter and P53 reporter luciferase vectors were subjected to DMF or oxaliplatin treatment for 24 h, and luciferase activity density was measured. (**B**) Western blotting of YAP signaling pathway activation in HCT8, SW480, HT29, and LoVo cells. (**C**) Western blotting of ERK signaling pathway activation in HCT8, SW480, HT29, and LoVo. (**D**) Western blotting of HCT8 (left) and SW480 (right) for ERK signaling pathway activation after oxaliplatin treatment for 0, 6, 24, and 48 h. (**D**) Western blotting of LoVo (left) and HT29 (right) for YAP signaling pathway activation after oxaliplatin treatment for 0, 6, 24, and 48 h. (**E**) Western blotting of HCT8 (left) and SW480 (right) for YAP signaling pathway activation after oxaliplatin treatment for 0, 6, 24, and 48 h. (**F**) Western blotting of LoVo (left) and HT29 (right) for YAP signaling pathway activation after oxaliplatin treatment for 0, 6, 24, and 48 h. (**G**) The YAP reporter luciferase vector was transiently transfected with oxaliplatin or bosentan treatment for 24 h, and luciferase activity density was measured. (**H**) EdU assay for cell proliferation after oxaliplatin (1 μM, 24 h), verteporfin (1 μM, 24 h), or bosentan (40 μM, 24 h) treatment. Representative images: Azide555 (red) or Azide488 (green) represents cell proliferation, and Hoechst 33342 (blue) represents the nucleus. Scale bar = 100 μm. Data are shown as mean ± SD of three replicates. **p < 0.01, *p < 0.05, ns, no significance

Oncogenic signaling pathways, including NF-κB [29], P53 [30], and YAP signaling [31] pathways have been implicated in mediating oxaliplatin resistance by regulating apoptosis, DNA damage repair, epithelial–mesenchymal transition, and the expression of drug efflux transporters. To investigate the involvement of these pathways in our model, we assessed their transcriptional activity using reporter assays in oxaliplatin-treated HCT8 and SW480 cells. Among the three pathways, only YAP reporter activity was significantly upregulated in both cell lines following oxaliplatin exposure (Fig. 5A), indicating selective activation of YAP signaling in response to chemotherapy.

To validate these findings, we performed Western blot analysis for phospho-YAP at Tyr357 (p-YAP Tyr357), a modification known to enhance YAP nuclear localization and transcriptional activity. Notably, p-YAP Tyr357 levels were markedly elevated in EDN1-high CRC cell lines (LOVO and HT29), whereas minimal induction was observed in EDN1-low lines (HCT8 and SW480) (Fig. 5B), further supporting the association between EDN1 expression and YAP activation. Similarly, increased ERK phosphorylation was observed in EDN1-high cells (Fig. 5C); however, time-course analysis following oxaliplatin treatment (0, 6, 24, 48 h) demonstrated sustained activation of the YAP pathway (Fig. 5E,F), while ERK signaling progressively declined (Fig. 5D). These results suggest that ERK may not play a direct role in oxaliplatin response, whereas YAP remains persistently active under chemotherapeutic stress. Consistently, dual-luciferase reporter assays revealed that oxaliplatin treatment significantly enhanced YAP transcriptional activity, which was effectively suppressed by Boesentan, a selective EDN1 receptor antagonist (Fig. 5G).

**Figure 6:**
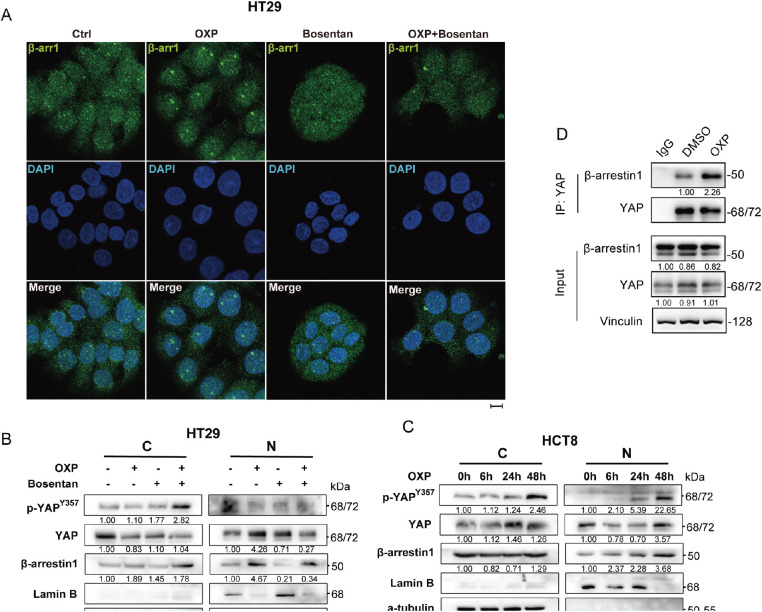
Chemoresistance of oxaliplatin is via the EDN1/β-arr1/YAP axis. (**A**) HT29 immunofluorescence staining for β-arr1 (green) and DAPI (blue) after 48 h treatment with oxaliplatin, bosentan, or a combination of the two drugs. Scale bars = 100 μm. (**B**) Western blot of cytoplasmic and nuclear YAP signaling pathways and β-arr1 of HT29, using tubulin and laminin B as controls for preparation purity. (**C**) Western blot of cytoplasmic and nuclear YAP signaling pathways and β-arr1 of HCT8, using tubulin and laminin B as controls for preparation purity. (**D**) Interaction of endogenous β-arr1 with endogenous YAP in colorectal cancer cells. Endogenous YAP was immunoprecipitated from HCT8 cells and its interaction with β-arr1 was detected by Western blot. The interaction between β-arr1 and YAP was detected after OXP (1 μM, 24 h) treatment. IgG was used as a negative control

**Figure A2:**
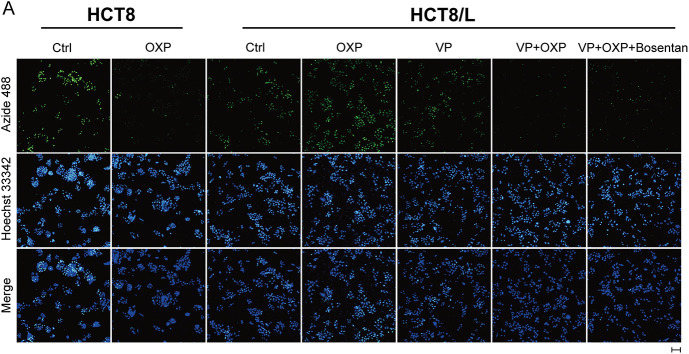

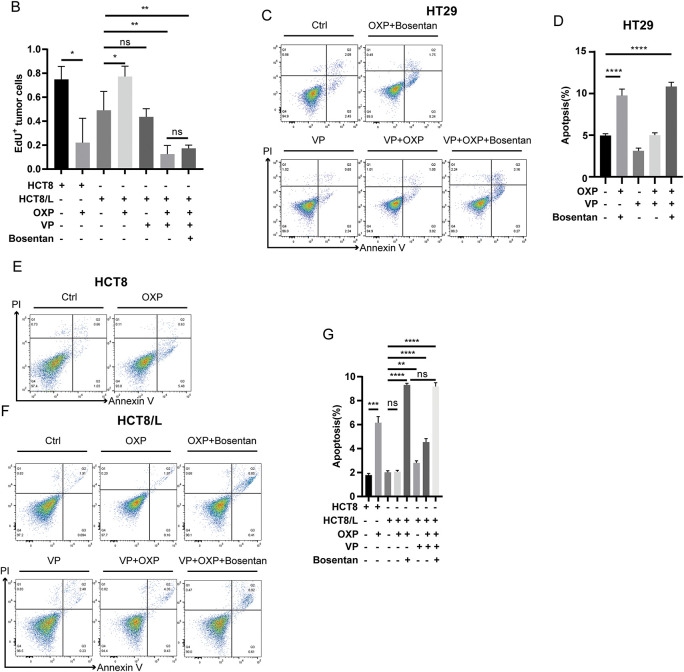
Oxaliplatin combined with verteporfin or bosentan had the same effect on cell proliferation and apoptosis. (**A**) EdU assay for cell proliferation after oxaliplatin (1 μM, 24 h), verteporfin (1 μM, 24 h) or bosentan (40 μM, 24 h) treatment. Representative images: Azide555 (red) or Azide488 (green) represents cell proliferation, and Hoechst 33342 (blue) represents the nucleus. Scale bar = 100 μm. (**B**) Quantitative analysis of EdU assay results. (**C**,**E**,**F**) Flow cytometry was used to evaluate verteporfin (0.5 μM, 12 h), bosentan (80 μM, 24 h) or oxaliplatin (10 μM, 48 h) treatment of cells in live cells (Annexin V^−^/PI^−^), early apoptotic cells (Annexin V^+^/PI^−^) and late apoptotic cells (Annexin V^+^/PI^+^) percentages. (**D**,**G**) Annexin V/PI staining quantitative analysis of HT29, HCT8 and HCT8/L. Data are shown as a flat ± SD for three replicates. ****p < 0.0001, ***p < 0.001, **p < 0.01, *p < 0.05, ns, no significance

To functionally confirm the role of YAP in EDN1-mediated oxaliplatin resistance, we conducted cell proliferation assays in oxaliplatin-resistant HCT8/L and HT29 cells following treatment with Boesentan or Verteporfin, a pharmacological inhibitor of YAP-TEAD interaction (Figs. 5H and A2A). Both Boesentan and Verteporfin significantly sensitized resistant cells to oxaliplatin, as evidenced by suppressed cell proliferation in combinatorial treatments compared to monotherapies (Figs. 6A and A2A,B). Apoptosis assays further demonstrated that the combination of oxaliplatin with either Boesentan or Verteporfin significantly increased apoptotic rates, whereas the addition of a third agent did not confer additional benefit (Fig. A2C–G). Collectively, these results demonstrate that EDN1 upregulation promotes oxaliplatin resistance in colorectal cancer via YAP pathway activation. Inhibiting YAP signaling restores sensitivity to oxaliplatin, suppresses proliferation, and enhances apoptosis, underscoring the therapeutic potential of targeting the EDN1-YAP axis to overcome chemoresistance in colorectal cancer.

### 3.5 Oxaliplatin Drives **β**-Arrestin1 Nuclear Translocation and Facilitates YAP Pathway Activation

**Figure A3:**
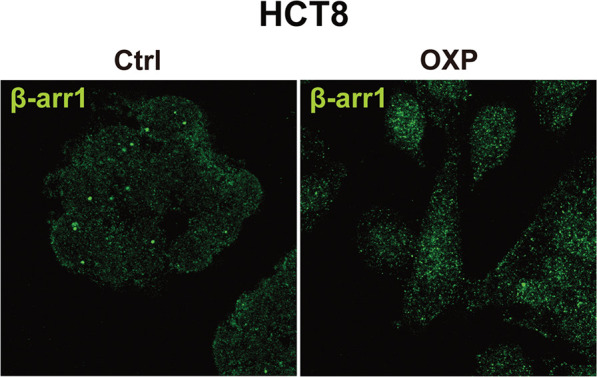

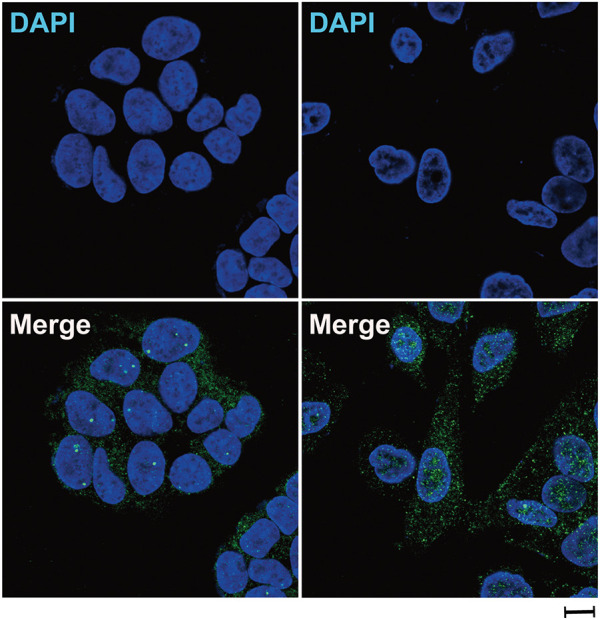
Oxaliplatin treatment induced β-arrestin1 nuclear translocation in CRC cells. Immunofluorescence staining of HCT8 for β-arrestin1 (green) and DAPI (blue) after treatment with 10 μM oxaliplatin for 48 h. Scale bar = 100 μm

ET-1 binding to ETAR activates downstream signaling by inducing receptor conformational changes and promoting β-arrestin1(β-arr1) recruitment [17,18]. β-arr1 functions as a multifunctional scaffold that mediates receptor endocytosis and trafficking [32–34]. Emerging studies have further shown that β-arrestin1 can translocate into the nucleus to modulate gene transcription [35,36]. Given these multifaceted roles, we hypothesized that EDN1 may contribute to oxaliplatin resistance by promoting β-arr1 nuclear translocation and its interaction with nuclear YAP. To explore this possibility, we first examined whether oxaliplatin alters the subcellular distribution of β-arr1. Immunofluorescence staining revealed that oxaliplatin treatment significantly increased nuclear localization of β-arr1 in HCT8 (Fig. A3) and HT29 cells (Fig. 6A). This nuclear accumulation was corroborated by subcellular fractionation and immunoblotting (Fig. 6B,C), which demonstrated a marked increase in β-arr1 in nuclear fractions, whereas cytoplasmic levels remained unchanged.

To further elucidate the mechanistic link between β-arr1 and YAP signaling, we performed Co-IP assays and confirmed a direct interaction between β-arr1 and YAP in CRC cells (Fig. 5D). Importantly, oxaliplatin treatment enhanced the association between β-arr1 and YAP, suggesting a functional partnership in the context of chemotherapeutic stress. YAP signaling was activated in both the cytoplasmic and nuclear compartments, whereas β-arr1 accumulation was predominantly nuclear and increased progressively in a time-dependent manner following oxaliplatin treatment. tin1 accumulation in the nucleus increased progressively after oxaliplatin exposure (Fig. 6C).

In HT29 cells, treatment with bosentan effectively attenuated oxaliplatin-induced β-arr1 nuclear accumulation (Fig. 6B), supporting the hypothesis that EDN1-ETAR signaling mediates this translocation event. Collectively, these findings demonstrate that oxaliplatin-induced overexpression of EDN1 promotes the nuclear translocation of β-arr1, thereby facilitating the activation of YAP signaling and contributing to chemoresistance in colorectal cancer.

### 3.6 Bosentan Enhances Oxaliplatin-Mediated Anti-Tumor Efficacy In Vivo

**Figure 7:**
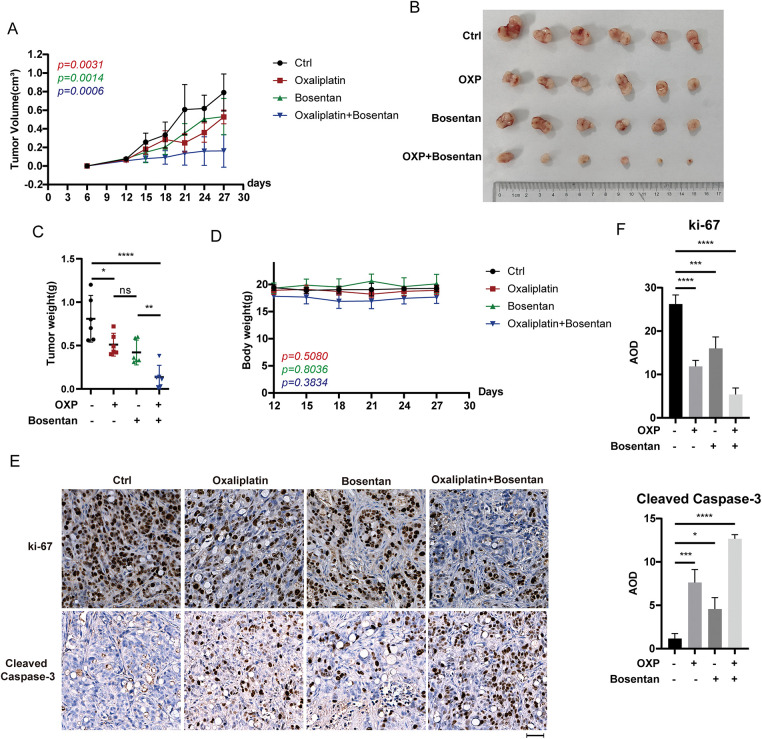
EDN1 inhibitor, in combination with oxaliplatin, slows tumor growth *in vivo*. (**A**) Nude mice were given a subcutaneous xenograft of 5 × 10^6^ cells. The administration was performed on day 12, and the growth of tumors in mice was plotted as a line graph (*n* = 6). (**B**) Pictures of subcutaneously implanted xenograft HT29 tumors (*n* = 6). (**C**) Weight of subcutaneously implanted HT29 tumors (*n* = 6). (**D**) Changes in body weight of mice after administration (*n* = 6). (**E**) Representative IHC analysis of mouse tumors using the anti-Ki67 and anti-cleaved caspase-3 antibodies. Scale bar = 50 μm. (**F**) Quantitative analysis of IHC. Data are shown as a mean ± SD for three replicates. ****p < 0.0001, ***p < 0.001, **p < 0.01, *p < 0.05, ns, no significance

To evaluate the therapeutic potential of EDN1 inhibition *in vivo*, we performed xenograft assays using HT29 colorectal cancer cells. Mice were randomized into four treatment groups receiving vehicle control, bosentan, oxaliplatin, or a combination of bosentan and oxaliplatin. Notably, combination treatment resulted in a significant reduction in tumor volume compared to either monotherapy or control (Fig. 7A–C), whereas bosentan or oxaliplatin alone failed to elicit a statistically significant anti-tumor effect. Importantly, no significant changes in body weight were observed across treatment groups (Fig. 7D), suggesting that the combination therapy was well tolerated and did not induce overt systemic toxicity.

Moreover, immunohistochemical analyses further revealed that tumors from the combination group exhibited markedly reduced expression of Ki-67, a proliferation marker, and increased cleaved caspase-3, indicative of enhanced apoptosis, relative to the control and monotherapy groups (Fig. 7E,F). These data indicate that the combined treatment not only suppresses tumor cell proliferation but also promotes apoptotic cell death *in vivo*. Collectively, our *in vivo* findings provide strong evidence that pharmacological blockade of EDN1 using bosentan enhances the anti-tumor efficacy of oxaliplatin and may represent a promising strategy to overcome oxaliplatin resistance in colorectal cancer.

## 4 Discussion

The emergence of chemoresistance in CRC represents a substantial obstacle to effective treatment, particularly in the context of platinum-based chemotherapies such as oxaliplatin. The activation of multiple anti-apoptotic pathways within an integrated signaling network is a critical factor in the development of this resistance. The study elucidates the ET-1/β-arr1/YAP signaling axis as a critical driver of oxaliplatin resistance in CRC, highlighting its translational potential for overcoming chemoresistance. The discovery that β-arr1 promotes YAP nuclear translocation and activates pro-survival gene transcription provides mechanistic insight into how cancer cells evade oxaliplatin-induced apoptosis. Notably, the feedback loop linking oxaliplatin to EDN1 overexpression underscores a self-reinforcing pathway that perpetuates resistance—a finding with direct clinical relevance, as it identifies EDN1 as a druggable target to disrupt this cycle.

The consistent elevation of EDN1 in oxaliplatin-resistant and inherently insensitive CRC cells, coupled with functional validation through genetic manipulation, positions EDN1 inhibition as a promising strategy to resensitize tumors. These observations align with emerging evidence implicating endothelin signaling in therapy resistance across cancers but extend its implications to CRC-specific oxaliplatin resistance. Future studies should prioritize translating these findings into clinical applications, such as combining EDN1 antagonists (e.g., bosentan) with oxaliplatin-based regimens. Additionally, validating this axis in patient-derived models and correlating EDN1/YAP activity with clinical outcomes in CRC cohorts will strengthen its prognostic and therapeutic relevance. Ultimately, this work bridges a critical knowledge gap in chemoresistance mechanisms and proposes an actionable combinatorial approach to improve CRC treatment efficacy.

The involvement of G protein-coupled receptors (GPCRs) in the activation of YAP signaling is well-established, with previous studies highlighting G protein-dependent pathways as key mediators [21,37]. Specifically, ET-1 has been shown to promote YAP/TAZ nuclear accumulation through G protein signaling in CRC cells with high ETAR expression [38]. However, our study provides novel insights by identifying β-arr1 as a mediator of ET-1-induced YAP nuclear localization in CRC cells. This finding aligns with reports in ovarian cancer, where β-arr1 has been shown to form complexes with YAP and P53 in the nucleus, contributing to apoptotic evasion [39]. Notably, we observed no significant changes in cytoplasmic β-arr1 levels following oxaliplatin or inhibitor treatment, while nuclear β-arr1 levels exhibited significant variability, supporting a role for nuclear β-arr1 in YAP activation [18]. These findings suggest that β-arr1-mediated nuclear signaling facilitates YAP/TAZ activation, promoting chemoresistance. However, further studies, including the assessment of YAP/TAZ activity following β-arr1 knockdown, are required to fully elucidate this mechanism.

Importantly, we addressed the mechanistic link between β-arr1 and YAP activation by demonstrating an oxaliplatin-induced increase in YAP reporter activity and in YAP phosphorylation at Tyr357. While the phosphorylation of YAP at Ser127, Ser397, and other LATS1/2-regulated residues is associated with YAP inactivation via cytoplasmic sequestration and proteasomal degradation, phosphorylation at Tyr357 represents a distinct regulatory event [40]. This site, targeted by Src-family kinases, has been shown to promote YAP nuclear localization and transcriptional activity [41]. Thus, the observed increase in p-YAP Tyr357 in response to oxaliplatin is consistent with YAP activation, rather than inhibition, and correlates with increased TEAD-driven transcription and pro-survival gene expression. This distinction emphasizes the importance of site-specific phosphorylation in determining YAP functional status and addresses concerns regarding canonical Hippo pathway-mediated inactivation. Nevertheless, further work using site-specific YAP mutants and nuclear/cytoplasmic localization assays will be required to fully delineate the context-dependent regulation of YAP by oxaliplatin.

To explore the therapeutic potential of targeting EDN1, the study employed bosentan, a well-characterized dual ETAR/ETBR antagonist, in both *in vitro* and *in vivo* models. Bosentan, which is approved for the treatment of pulmonary hypertension and systemic sclerosis, has been shown to competitively inhibit ETAR and ETBR [42,43]. Our results demonstrate that bosentan effectively disrupts the ET-1/β-arr1/YAP signaling network, thereby enhancing oxaliplatin-induced apoptosis in resistant CRC cells. *In vivo*, combined treatment with bosentan and oxaliplatin significantly inhibited tumor growth in HT29 xenograft models compared to either treatment alone, indicating a synergistic effect that may be leveraged to overcome chemoresistance in clinical settings.

Given that bosentan targets both ETAR and ETBR, and considering the emerging role of ETBR in modulating tumor immunity [44,45]. Our findings raise the possibility that bosentan’s anti-tumor effects may involve immune-mediated mechanisms. This warrants further investigation to elucidate the interplay between EDN1 signaling and the tumor immune microenvironment. Currently, bosentan is not utilized in the context of chemotherapy resistance. Our data suggest that combining bosentan with oxaliplatin not only promotes apoptosis but also represents a promising therapeutic strategy for enhancing the efficacy of chemotherapy. This combinatorial approach may inform the development of novel regimens aimed at improving outcomes for CRC patients with refractory disease.

While the study provides novel insights into the EDN1/β-arr1/YAP axis in oxaliplatin resistance, several limitations should be acknowledged. First, the mechanistic exploration primarily relied on *in vitro* cell line models (HCT8/L, HCT116/L) and selected CRC cell lines with inherent sensitivity differences (HT29, HCT8). While these models are widely used, they may not fully recapitulate the complexity of tumor-microenvironment interactions or heterogeneous chemoresistance mechanisms observed in clinical CRC patients. Validating these findings in patient-derived organoids, xenografts, or clinical cohorts would strengthen their translational relevance. Second, the study focused on the EDN1-YAP axis but did not comprehensively address potential crosstalk with other resistance-related pathways, such as autophagy, DNA damage repair, or immune evasion mechanisms. Whether these pathways synergize with or counteract the EDN1/β-arr1/YAP signaling remains unclear, leaving room for further mechanistic dissection. Lastly, the clinical correlation between EDN1/YAP activity and oxaliplatin resistance in human CRC tissues was not directly assessed. Future studies should integrate multi-omics data from clinical samples to establish the prognostic value of this axis and identify patient subgroups most likely to benefit from EDN1-targeted therapies.

## 5 Conclusions

Our study identifies the EDN1/β-arr1/YAP axis as a critical mediator of oxaliplatin resistance in CRC. Pharmacological inhibition of this pathway using agents such as bosentan significantly enhances the therapeutic efficacy of oxaliplatin and offers a promising strategy to overcome chemoresistance. These findings provide a strong rationale for further clinical investigation into EDN1-targeted therapies and support their potential translational application in the treatment of colorectal cancer.

## Data Availability

The data that support the findings of this study are available from the corresponding author, Hong Zhang, upon reasonable request.

## Nomenclature

CRC: Colorectal cancer
OXP: Oxaliplatin
β-arr1: β-arrestin1
STR: Short Tandem Repeat
BSA: Bovine Serum Albumin
EDTA: Ethylene Diamine Tetraacetic Acid
DAB: 3,3^′^-Diaminobenzidine
shRNA: Short hairpin RNA
Log_2_FC: Log_2_ Fold Change
TCGA: The Cancer Genome Atlas
GEO: Gene Expression Omnibus
GPCR: G-protein-coupled receptors
ET: Endothelin
5-FU: 5-fluorouracil
YAP: Yes-associated protein
CST: Cell Signaling Technology
